# Increased Regular Season Soft Tissue Injury Rates in National Football League (NFL) Players May Be Associated With the Canceled 2020 NFL Preseason Due to COVID-19

**DOI:** 10.7759/cureus.24674

**Published:** 2022-05-02

**Authors:** Salvatore Sclafani, Nicholas Frane, Tyler J Humphrey, Joseph Miceli, Robert Trasolini

**Affiliations:** 1 Department of Orthopedic Surgery, Northwell Health Plainview Hospital, Plainview, USA; 2 Orthopedic Surgery, The Center for Orthopedic Research and Education (CORE) Institute, Phoenix, USA; 3 Department of Orthopedic Surgery, Massachusetts General Hospital, Harvard Medical School, Boston, USA; 4 Department of Orthopedic Surgery, Jersey City Medical Center, Jersey City, USA

**Keywords:** soft tissue injury, covid-19, athlete workload, sports injury, nfl

## Abstract

Introduction

The purpose of this study is to evaluate the rates of regular season soft tissue injuries in National Football League (NFL) players during the 2020 season, which had a canceled preseason due to the COVID-19 pandemic.

Methods

This study retrospectively reviewed the injury rates of the 2020-2021 NFL regular season in comparison to the 2018-2019 NFL regular season using publicly available injury data. The focus of our analysis was comparing the following soft tissue injuries: hamstring, groin, calf, quadriceps, thigh, knee - anterior cruciate ligament (ACL), pectoral, and Achilles. The week of injury occurrence, duration of injury in weeks, position of the injured player, and age of the NFL player at injury were obtained. Injury rates were calculated per 1000 athletic exposures with 95% confidence intervals (CIs). A chi-square test and Student’s t-test were utilized as appropriate.

Results

There were 1370 total injuries in the 2018-2019 regular NFL season and 2086 total injuries reported in the 2020-2021 regular NFL season. The total number of injuries per 1000 athletic exposures was significantly higher in the 2020-2021 NFL season compared to the 2018-2019 NFL season (88.57 versus 58.17, p < 0.001). The rates of injuries per 1000 athletic exposures for hamstring (9.98 versus 5.31, p = 0.043), groin (5.56 versus 2.46, p = 0.007), calf (4.08 versus 1.61, p = 0.006), quadriceps (2.00 versus 0.72, p = 0.030), and thigh (1.23 versus 0.30, p = 0.012) injuries were significantly higher in the 2020-2021 regular NFL season compared to the 2018-2019 NFL regular season.

Conclusions

The 2020-2021 NFL season had a significantly higher incidence of soft tissue injuries compared to the 2018-2019 regular NFL season, which may have been associated with the absent preseason due to the COVID-19 pandemic and an abrupt increase in the athletic workload of players.

## Introduction

The SARS-CoV-2 (COVID-19) pandemic created a unique environment for athletes in the National Football League (NFL) and the opportunity to evaluate injury rate data for the 2020-2021 regular NFL season compared to a previous “normal” season. Usually, the regular NFL season is preceded by two weeks of training camp and a four-week preseason with weekly games against other teams. This serves to prepare NFL athletes for the demands of the season, with the four preseason games serving as a transition to acclimate players to the speed and intensity of the regular season. However, due to the rising number of cases of SARS-CoV-2 in the United States of America (USA), the NFL canceled the 2020 preseason and implemented several new restrictions on training camps [1,2].

In comparison to previous NFL preseasons, in the 2020 preseason, there was a much longer period of time prior to the regular season focused on cardiovascular conditioning and other non-football activities. As a result, players were not involved in on-field workouts, team practices, and other organized team activities. Furthermore, many players were unable to access gyms or their usual training equipment [2]. Historically, the only similar situation was the 2011-2012 NFL lockout (from March 11 to July 25, 2011). This lockout had also resulted in an abnormal off-season that left players without the usual access to their teams’ healthcare providers, strength and conditioning professionals, and coaches, which was associated with an increased incidence of Achilles tendon ruptures in NFL players in comparison with previous years [3].

Athletes who are subject to large fluctuations in the amount of soft tissue loadbearing sustained over a short period of time (e.g., NFL player starting the regular season without a proper preseason) may be subject to increased microdamage to soft tissues, altered kinematics during high-intensity gameplay, and decreased joint stability, leading to an increased incidence of soft tissue injuries [4-8]. Identifying modifiable factors that may contribute to increased soft tissue injuries could help mitigate these injuries, which has implications for athletes, teams, and medical personnel. We sought to explore how an abbreviated training period prior to regular season play might have been associated with soft tissue injury incidence.

To our knowledge, previous studies have not looked specifically at soft tissue injuries in NFL athletes over this period of time. Recently, there has been some literature evaluating the effects of the canceled preseason due to the COVID-19 pandemic on the 2020-2021 NFL season. These studies have either looked at comparisons of the total numbers of injuries (which included head injuries, bony injuries, and fractures), only looked at a select number of weeks at the beginning of the season, or focused on position-level changes in injury rates [9-11]. As previous evidence has suggested that extended periods of stoppage of professional training with an abrupt resumption of full activity can place athletes at an increased risk for soft tissue deconditioning injury, in particular, we hypothesized that the training limitations set in response to the COVID-19 pandemic could be associated with an increased incidence of soft tissue injuries during the NFL 2020-2021 season.

## Materials and methods

NFL player injury data from the 2020-2021 regular NFL season and from the 2018-2019 regular NFL season were obtained from publicly available NFL injury reports (http://www.nfl.com/injuries). For players who missed games due to injury, we confirmed an absence of statistics for the games that they were ruled out. Each injury data point was defined as any player appearing on the injury report with an anatomic location and game status designation that was unique from the previous week’s injury report. Player injuries listed with the same anatomic location and game status for consecutive weeks were considered as one injury. However, the number of weeks the injury persisted was documented as a surrogate for the severity of the injury. Injuries were classified based on the anatomic location of the injury, impact of the injury on playing status (“out,” “questionable,” “doubtful,” or “injured reserve” (IR)), position of the injured player, age of the injured player, week in the season in which the injury occurred, and duration (in weeks) of injury. To determine the duration of injury for players, the week in which players were placed on IR (or listed as doubtful, out, etc.) was subtracted from the week they returned from the injury (or from week 17 if they remained out for the remainder of the season).

Eight anatomic locations of soft tissue injury were selected as the focus of our analysis: hamstring, groin, calf, quadriceps, thigh, knee - anterior cruciate ligament (ACL), pectoral, and Achilles. All other injury data beyond the eight select soft tissues as the focus of our analysis (e.g., stingers, fractures, and concussions) were accounted for in our number of “total injuries” in each season. We excluded all preseason and postseason injuries and excluded “illness” as the documented reason for injury; this exclusion was applied to both the injury totals we defined and from any analysis in our study.

Injury reporting and statistical analysis

The concept of athletic exposures is frequently used when analyzing the epidemiology of athletic injuries and was used to quantify the injury rate for each season [12]. Athletic exposures were calculated in a similar manner to Stapleton et al. as follows: 46 active players play on each team per game, leading to 92 players at risk for each NFL game. The total number of games in the regular NFL season is 256. With 92 players at risk multiplied by 256 games, this gives 23,552 yearly athletic exposures [13]. Using 23,552 yearly athletic exposures, we calculated the regular season injury rate per 1000 athletic exposures for each season. This method has also been described in other studies examining database-level athletic injury statistics [14]. The calculation for season injury rate per 1000 athletic exposures was the total number of injuries for a select injury anatomic location multiplied by 1000 and then divided by 23,552.

Descriptive statistics such as means and percentages were calculated for the injury rates of the eight select soft tissue injuries in each season. For categorical variables greater than 10 data points, a χ2 test was performed; for variables with less than 10 data points, Fisher’s exact test was performed to assess for differences between groups. A Student’s t-test was performed for continuous variables. Furthermore, 95% confidence intervals (CIs) are reported for injuries per 1000 athletic exposures as described by Knowles et al. [14]. P-values less than 0.05 were considered significant. Statistical analyses were performed using IBM SPSS Statistics for Windows version 26 (IBM Corp., Armonk, NY, USA).

## Results

We identified 1370 injuries in the 2018-2019 regular NFL season and 2086 injuries reported in the 2020-2021 regular NFL season. There were no significant differences in player age for any soft tissue injury across the two seasons (data not shown). Table 1 portrays the total number of the eight soft tissue injuries of interest for each season, regardless of injury statistics (e.g., out, doubtful, questionable, and injured reserve). The total (unadjusted) numbers of the eight soft tissue injuries of our study’s focus are listed adjacent to the injuries per 1000 athletic exposures (Table 1). The adjusted rates of injuries per 1000 athletic exposures for hamstring (9.98 versus 5.31, p = 0.043), groin (5.56 versus 2.46, p = 0.007), calf (4.08 versus 1.61, p = 0.006), quadriceps (2.00 versus 0.72, p = 0.030), and thigh (1.23 versus 0.30, p = 0.012) injuries were significantly higher in the 2020-2021 regular NFL season compared to the 2018-2019 NFL regular season. The total number of injuries per 1000 athletic exposures in the 2020-2021 regular season, inclusive of both the eight soft tissue injuries listed in Table 1 and all other reported injuries during the seasons (e.g., concussions and fractures), was significantly higher than the total number of injuries for 1000 athletic exposures in the 2018-2019 regular season as well (88.57 versus 58.17, p < 0.001).

**Table 1:** Average Incidence of the Eight Select Soft Tissue Injuries in Each Respective Season, Including All Injury Designations NFL: National Football League, ACL: anterior cruciate ligament, CI: confidence interval *p-value for comparison between columns comparing athletic exposures Bolded p-values reflect significance (p < 0.05).

	2018-2019 NFL Season Unadjusted Injury Numbers	2020-2021 NFL Season Unadjusted Injury Numbers	2018-2019 NFL Season Injuries per 1000 Athletic Exposures (95% CIs)	2020-2021 NFL Season Injuries per 1000 Athletic Exposures (95% CIs)	P-Value*
Hamstring	125	235	5.31 (5.12-5.50)	9.98 (9.72-10.24)	0.043
Groin	58	131	2.46 (2.33-2.59)	5.56 (5.37-5.76)	0.007
Calf	38	96	1.61 (1.51-1.72)	4.08 (3.91-4.24)	0.006
Quadriceps	17	47	0.72 (0.65-0.79)	2.00 (1.88-2.11)	0.030
Thigh	7	29	0.30 (0.25-0.34)	1.23 (1.14-1.32)	0.012
Knee - ACL	16	28	0.68 (0.61-0.75)	1.19 (1.10-1.28)	0.251
Pectoral	10	22	0.42 (0.37-0.48)	0.93 (0.85-1.01)	0.165
Achilles	17	17	0.72 (0.65-0.79)	0.72 (0.65-0.79)	0.214
Total injuries in the season	1370	2086	58.17 (57.53-58.80)	88.57 (87.79-89.35)	<0.001

Table 2 compares the average week of injury occurrence and the average duration of injury of the eight select soft tissue injuries between the 2018-2019 and 2020-2021 regular NFL seasons. Interestingly, the average duration of injury of hamstring, groin, calf, quadriceps, and thigh injuries was significantly shorter in the 2020-2021 regular NFL season compared to the 2018-2019 regular NFL season. We were not able to detect a significant difference in terms of the average week of injury occurrence between the two seasons (Table 2).

**Table 2:** Comparison of the Average Week Injured and Average Duration of Injury (in Weeks) for the Eight Select Soft Tissue Injuries Between the 2018-2019 NFL Season and the 2020-2021 NFL Season NFL: National Football League, ACL: anterior cruciate ligament ± represents 1 standard deviation. Bolded p-values reflect significance (p < 0.05).

	2018-2019 NFL Season	2020-2021 NFL Season	P-Value
Hamstring injuries			
Average week injured	6.73 ± 4.84	7.64 ± 4.92	0.125
Average duration of injury	3.504 ± 3.57	2.06 ± 1.91	0.001
Groin injuries			
Average week injured	7.28 ± 4.41	8.39 ± 4.77	0.138
Average duration of injury	3.84 ± 4.89	1.67 ± 1.82	0.001
Calf injuries			
Average week injured	8.16 ± 4.72	9.20 ± 5.35	0.296
Average duration of injury	2.42 ± 1.47	1.84 ± 1.92	0.096
Quadriceps injuries			
Average week injured	8.82 ± 5.64	7.71 ± 4.71	0.435
Average duration of injury	3.35 ± 3.21	1.78 ± 1.82	0.017
Thigh injuries			
Average week injured	5.29 ± 2.63	9.07 ± 5.06	0.065
Average duration of injury	4.71 ± 4.68	1.41 ± 0.91	0.001
Knee - ACL injuries			
Average week injured	6.53 ± 2.90	7.58 ± 5.07	0.470
Average duration of injury	11.4 ± 2.85	10.71 ± 5.13	0.623
Pectoral injuries			
Average week injured	8.56 ± 4.25	5.73 ± 4.68	0.153
Average duration of injury	5.55 ± 4.95	2.8 ± 3.05	0.062
Achilles injuries			
Average week injured	9.76 ± 5.39	8.31 ± 5.34	0.443
Average duration of injury	5.64 ± 4.61	8.17 ± 6.50	0.199

Table 3 provides a breakdown of which positional players accounted for the eight soft tissue injuries at the focus of our study between the 2018-2019 and 2020-2021 regular NFL seasons.

**Table 3:** Positional Stratification of the Numbers of Soft Tissue Injuries Between the 2018-2019 and 2020-2021 NFL Seasons NFL: National Football League, ACL: anterior cruciate ligament Position combinations are as follows: lineman includes offensive line, defensive line, defensive end, guard, and center; receiving includes tight end, wide receiver, and slot receiver; and special teams include punter and kicker.

2020-2021 NFL Season							
Injury location	Quarterback	Running back/fullback	Linemen	Cornerback and safeties	Linebackers	Receiving	Special teams
Hamstring	3	25	26	79	34	63	0
Groin	0	5	41	39	13	17	7
Calf	2	6	34	19	14	16	1
Quadriceps	0	10	2	17	10	5	1
Thigh	0	3	5	9	1	11	0
Knee - ACL	1	2	6	8	2	5	0
Pectoral	0	0	10	4	14	0	0
Achilles	0	0	12	18	0	3	0
2018-2019 NFL Season							
Injury location	Quarterback	Running back/fullback	Linemen	Cornerback and safeties	Linebackers	Receiving	Special teams
Hamstring	0	14	8	23	29	31	1
Groin	0	4	8	23	6	12	4
Calf	0	2	21	5	5	5	0
Quadriceps	0	1	5	3	1	6	1
Thigh	0	0	0	3	2	1	1
Knee - ACL	1	4	2	2	2	4	0
Pectoral	0	0	5	3	2	0	0
Achilles	0	1	4	3	0	9	0

## Discussion

Our study focused on investigating the association between the canceled 2020-2021 NFL preseason and soft tissue injury rates in the 2020-2021 regular NFL season in comparison to a previous NFL season with a full preseason. We found that hamstring, groin, calf, quadriceps, thigh, and total injury counts were significantly higher in the 2020-2021 regular NFL season compared to the 2018-2019 regular NFL season. In addition, the average duration of specific soft tissue injuries, namely, hamstring, groin, quadriceps, and thigh injuries, was significantly shorter in the 2020-2021 season compared to the 2018-2019 season. Notably, both the unadjusted and adjusted rates of pectoral, knee - ACL, and Achilles injuries were not significantly different between the 2020-2021 season and the 2018-2019 season (Table 1). For pectoral and knee - ACL injuries, in particular, the rates of injury were almost double (0.93 versus 0.42, and 1.19 versus 0.68, respectively) in the 2020-2021 season compared to the 2018-2019 season, while the number of Achilles injuries was the same (0.72) between the seasons. There are two possible explanations for this. First, these injuries are relatively less common than the other soft tissue injuries we analyzed; therefore, the data may have been underpowered to detect a significant difference. Second, pectoral, knee - ACL, and Achilles injuries are more likely the consequence of rare or unusual mechanisms instead of being associated with a decreased preseason training period [15].

In terms of position-level changes in injury incidence, similar to Bailey et al. [9], we found an increased number of defensive backfield players with injuries in the 2020-2021 season; however, we did not perform statistics on this finding as it was not the focus of our study. We attempted to account for player age as a potential confounder in our injury incidence analysis and found no statistical differences in the ages of players who sustained soft tissue injuries between the 2020-2021 and 2018-2019 seasons. Overall, these findings support our hypothesis that the absence of preseason is associated with a higher rate of soft tissue injuries. In light of these findings, the possible benefits of the NFL preseason and training camp should be promoted by league officials to mitigate the risk of injuries to athletes in the regular season.

The significance of preseason training, load management, and rapid changes in load

The purpose of preseason training is to prepare athletes for the physical demands of the upcoming season. In the absence of this period of physical conditioning, injuries are more likely to occur [16-18]. Our study’s findings are similar to recent research across multiple professional sports categories that suggest that rapid increases in athletic load are associated with increased risk for soft tissue injuries, rather than injury risk stemming from solely “absolute increases” in athletic load throughout a season [19-25]. For example, large changes in the load of athletic activity on a weekly basis appear to significantly increase the injury risk in rugby players [4]. Hulin et al. described that increases in the acute workload of Australian rugby players in relation to their chronic workload predicted soft tissue injury to a greater extent than players who maintained high workloads throughout the season [26]. These findings make sense physiologically, as it takes time for significant soft tissue adaptations to occur, which protect athletes from injury during high-impact athletic activity [27].

We suspect that the absent preseason of the 2020-2021 NFL season due to the COVID-19 pandemic created an abrupt rise in the “acute workload” of players who entered a rigorous NFL season, as these players who had no official preseason had a “chronically lowered workload” and did not have time to appropriately increase their athletic workload gradually during a normal preseason. Our suspicion is supported by research from Demir et al., who studied 30 professional soccer players subject to an eight-week COVID-19 quarantine. The authors found that this period of confinement led to a decrease in hamstring eccentric strength, a decrease in posterior chain flexibility in the legs, and an increase in hamstring injury incidence shortly after these players returned to professional soccer training [28]. While these findings are not unexpected from a biological perspective, understanding the population-level consequences of the lack of preseason training in high-intensity sports is necessary in order to develop league policies that encourage consistent athlete training and follow strict infection control guidelines.

Corollaries to the NFL lockout

A similar situation to the COVID-19 pandemic occurred prior to the initiation of the 2011-2012 NFL season, in which a lockout took place (from March 11 to July 25, 2011). This resulted in an abnormal off-season that also left players without normal access to their teams’ healthcare providers, strength and conditioning professionals, and coaches. A study by Myer et al. analyzed the effects of the lockout and rapid transition from the start of training camp to the initiation of preseason competition, which occurred over a period of 17 days. The authors reported 10 Achilles tendon injuries occurring over the first 12 days of training camp with two additional injuries occurring in the following 17 days [3]. Of note, 31 Achilles tendon ruptures were recorded between the 1997 and 2002 NFL seasons, with an average of five per year (35% occurred during the preseason and 65% during the regular season). The number of Achilles tendon tears in NFL players during the preparation for the 2011-2012 regular season (15 days of training camp and two weeks of preseason) exceeded all previously reported numbers of Achilles tendon ruptures that normally occurred over an entire season [3]. Interestingly, we did not find a significant difference in the numbers of Achilles injuries between the 2020-2021 regular NFL season and the 2018-2019 regular NFL season (17 versus 17 unadjusted Achilles injuries) (Table 1). It is possible that factors outside of an absent NFL preseason accounted for our study’s inability to detect a difference. Regardless, it is concerning that the total number of Achilles injuries in the two NFL seasons of our study period had approximately a threefold increase compared to the 1997-2002 NFL seasons.

Age as a possible confounder

The COVID-19 lockout also presented the opportunity to evaluate the effects of a rapid transition to the high demands of the NFL on younger, inexperienced players. While the COVID-19 lockout prevented all players from the opportunity to appropriately prepare for the upcoming season, it may be expected that a younger player, who is unfamiliar with the physical demands of the NFL and not as well-positioned financially to adapt their training regimen, would be more susceptible to injuries [29].

Myer et al. reported that of the 10 Achilles tendon ruptures that occurred during the 2011 post-lockout training camp, five occurred in rookies. In addition, the average NFL experience for all 12 players with Achilles tendon ruptures was only 1.4 years [3]. In comparison, Parekh et al. reported that 31 players who sustained an Achilles tendon rupture during the 1997-2002 NFL seasons (which included standard preseason training) were, on average, in the NFL for six years [30]. Furthermore, the average age of the players who sustained an Achilles tendon rupture in this study was approximately 29, whereas the average age of NFL players who sustained an Achilles tendon rupture in the 2011-2012 season was 23.9 years [3,30]. Our study found no significant differences in player age for any injury across the two seasons. Given this finding and that most active NFL players do not have any medical comorbidities placing them at increased risk for soft tissue injury, we suspect that the most likely cause for an increase in soft tissue injury incidence in the 2020-2021 NFL regular season compared to the 2018-2019 regular NFL season was the lack of preseason training and increased acute/chronic workload ratio experienced by players.

Limitations

This study should be interpreted in the context of its limitations. First, the integrity of the data that we obtained from public injury records is reliant on the accurate reporting of the teams that upload the injury reports. Nevertheless, as we obtained official injury reports, we believe that we have the most accurate injury reporting data available. Published injury data rarely provided the extent of the injury beyond what could be extrapolated by an injury’s duration in weeks or a player being designated to “out” or “injured reserve.” Second, the goal of this study is not to identify causality between the COVID-19 pandemic and an increased soft tissue injury incidence; rather, its goal is to identify a potential association that may serve as grounds for future prospective and retrospective studies that can evaluate outcomes associated with a shortened preseason.

Our study focuses on population-level changes in injury rates, which were obtained from an NFL injury database; thus, we are not able to report on the objective measures of deconditioning, and we are unable to make any statements about individual risk factors for soft tissue injury in our patient cohort. While we suspect that the suspension of the preseason during the 2020-2021 NFL year led to player deconditioning and subsequent injury, it is also possible that numerous other factors beyond the scope of this study were associated with each player’s injury, such as heterogeneity in return-to-play protocols for each team. We were also not able to account for any variation in the rest period between games that existed, as this could possibly explain changes in injury incidence as well. Furthermore, while it is possible that there are confounding variables relating to injury risk that we did not account for in our study, the majority of the NFL population has exceptional physical health, and any changes in year-to-year injury rates are likely due to physical conditioning prior to live gameplay or due to chance during actual gameplay.

## Conclusions

The 2020-2021 NFL season, with an absent preseason due to the COVID-19 pandemic, was characterized by a significantly higher incidence of hamstring, groin, calf, quadriceps, and thigh injuries compared to the 2018-2019 regular NFL season. Although we are unable to infer causality, we suspect that an increase in the acute/chronic workload ratio of the NFL athletes led to this increase in injury incidence. Our study suggests that NFL athletes may benefit from training policies that focus on continuing a normal preseason or consistent training regimen in accordance with established infection control guidelines, in order to limit both soft tissue injuries and infectious cases. Future studies should be performed to identify objective risk and preventative factors for soft tissue injury in athletes who are subject to shortened official preseason training before a full regular season.
